# Comparison of the vasodilator responses of isolated human and rat middle meningeal arteries to migraine related compounds

**DOI:** 10.1186/1129-2377-15-22

**Published:** 2014-04-23

**Authors:** Gustaf Grände, Sieneke Labruijere, Kristian Agmund Haanes, Antoinette MaassenVanDenBrink, Lars Edvinsson

**Affiliations:** 1 Department of Medicine, Institute of Clinical Science, Lund University, 221 84 Lund, Sweden; 2 Department of Internal Medicine, Division of Vascular Medicine and Pharmacology, Erasmus MC Rotterdam, PO Box 2040, 3000 CA Rotterdam, The Netherlands; 3 Department of Clinical Experimental Research, Copenhagen University Hospital, Glostrup, Denmark

**Keywords:** Vasodilation, Middle meningeal artery, Migraine, CGRP

## Abstract

**Background:**

Migraine attacks occur spontaneously in those who suffer from the condition, but migraine-like attacks can also be induced artificially by a number of substances. Previously published evidence makes the meninges a likely source of migraine related pain. This article investigates the effect of several vasodilators on meningeal arteries in order to find a connection between the effect of a substance on a meningeal vessel and its ability to artificially induce migraine.

**Methods:**

A myograph setup was used to test the vasodilator properties of the substances acetylcholine (ACh), sodium nitroprusside (SNP), sildenafil, prostaglandin E_2_ (PGE_2_), pituitary adenylate cyclase activating peptide-38 (PACAP-38), calcitonin gene-related peptide (CGRP) and NaCl buffer on meningeal arteries from human and rat. An unpaired t-test was used to statistically compare the mean E_max_(%) at the highest concentration of each substance to the E_max_(%) of NaCl buffer.

**Results:**

In the human experiments, all substances except PACAP-38 had an E_max_ (%) higher than the NaCl buffer, but the difference was only significant for SNP and CGRP. For the human samples, clinically tested antimigraine compounds (sumatriptan, telcagepant) were applied to the isolated arteries, and both induced a significant decrease of the effect of exogenously administrated CGRP. In experiments on rat middle meningeal arteries, pre-contracted with PGF_2α_, similar tendencies were seen. When the pre-contraction was switched to K^+^ in a separate series of experiments, CGRP and sildenafil significantly relaxed the arteries.

**Conclusions:**

Still no definite answer can be given as to why pain is experienced during an attack of migraine. No clear correlation was found between the efficacy of a substance as a meningeal artery vasodilator in human and the ability to artificially induce migraine or the mechanism of action. Vasodilatation could be an essential trigger, but only in conjunction with other unknown factors. The vasculature of the meninges likely contributes to the propagation of the migrainal cascade of symptoms, but more research is needed before any conclusions can be drawn about the nature of this contribution.

## Background

The dura mater and its vasculature, the middle meningeal artery (MMA) and the venous system have for decades been central to many hypotheses aimed to explain migraine pathophysiology [1,2]. Ray and Wolff showed in the 1940s that direct stimulation of the dura mater may result in headache with migraine-like qualities [3]. Further research during the last two decades has demonstrated that sensitization and local inflammation in the dura mater can be elicited by various agents and stimulation paradigms [4]. The local administration of an “inflammatory soup” or the direct stimulation at different parts of the dura mater may result not only in vasoactive responses but sometimes also in mast cell degranulation and in plasma extravasation. Histamine, a major amine released from mast cells, is known to induce a migraine-like pain upon infusion into test subjects previously diagnosed with migraine [5]. Some migraine-associated symptoms, including the characteristic pain, could therefore stem from the activation of meningeal mast cells and aseptic inflammation initiated by histamine release [6]. However, local administration of calcitonin gene-related peptide (CGRP), which is a sensory neuropeptide in the dura mater and a known migrainogenic substance [7], results only in vasodilatation [8]. Despite the fact that mast cells have CGRP receptors [9,10], no activation or sensitization of meningeal nociceptors could be detected upon topical or systemic administration of this compound in rats [8].

Apart from CGRP and histamine, other substances that upon intravenous infusion can provoke migraine-like attacks in humans previously diagnosed with migraine are prostaglandin E_2_ (PGE_2_), pituitary adenylate cyclase activating peptide-38 (PACAP-38), epoprostenol (prostacyclin, PGI_2_), sildenafil and nitric oxide (NO) [11-15]. The only common factor among these substances is their vasodilator properties, but still there exist several other vasodilators which do not induce migraine-associated symptoms upon infusion. Vasoactive intestinal polypeptide (VIP) and carbachol, both associated with the parasympathetic system, are noted examples, although their ability to dilate meningeal arteries *in vivo* remains to be verified [16,17]. This shows that factors apart from the vasodilatation have to be taken into consideration. There is still controversy regarding whether vasodilatation directly contributes to any of the migraine-related symptoms or whether this is just a side-phenomenon. Several hypotheses exist that downplay the role of vasodilatation, including the observation of prodromal aura symptoms preceding any activity in the vasculature [18], that the vasoconstrictor triptan drugs are not an effective treatment in all migraine patients and that the extravascular tissue surrounding the dural arteries could be too rigid to allow for any significant expansion of the vessel diameter [19].

We have recently investigated the vasoactive effects of several substances on cerebral arteries from human and rat *in vitro* in order to find common denominators that would allow us to understand more about the possible factors behind the migraine-associated symptoms [20]. However, cerebral arteries are different from meningeal arteries regarding several morphological aspects, including receptor expression, anatomical origin (internal carotid artery (ICA) versus external carotid artery (ECA)) and the lack of a blood–brain barrier (BBB) in the meningeal arteries [21]. Nevertheless, both vascular regions receive sensory input from the first division of the trigeminal ganglion [22]. Therefore it is possible that the two artery types contribute to migraine differently if there indeed is a vascular component.

The aim of the present study is to evaluate whether there is a connection between the ability of a substance to dilate meningeal arteries and the previously reported migrainogenic properties of each substance. It is useful to verify whether vasodilatation of the meningeal arteries indeed is the trigger for the migraine-like pain triggered by these substances. Since the meningeal arteries lack BBB properties, systemic drugs are freely diffusible to endothelial and smooth muscle receptors to elicit a vasomotor response. A secondary goal of this study is to compare the functional aspect of the middle meningeal artery (MMA) between the species because rat is often used in mechanistic studies due to the difficulty in obtaining human tissue samples.

## Methods

The experimental protocol (M11104) was approved by the Animal Protocol Review committee at the University of Lund. All human artery procedures were carried out strictly according to national laws and guidelines and approved by the Ethical Committee at the University of Lund (LU-818-01) and the local Ethics Committee at the Erasmus MC, Rotterdam.

### Obtaining the MMA

A. Fresh samples of MMA were obtained from the meninges of male Sprague–Dawley rats by the following procedure. The rats were anesthetized using CO_2_ and decapitated. The cranium was opened from the top and the brain removed, which exposed the dura mater. The cranium and dura mater were divided sagitally and each half was placed in a buffer solution composed of NaCl 119 mM, NaHCO_3_ 15 mM, KCl 4.6 mM, MgCl_2_ 1.2 mM, NaH_2_PO_4_ 1.2 mM, CaCl_2_ 1.5 mM and glucose 5.5 mM. The dura mater was carefully separated from the cranium and moved to a Petri dish, where it was submerged in buffer solution, stretched and suspended by needles. The MMA was removed from the dura mater by a dissecting microscope, cut into segments 1–2 mm long that were placed in parallel tissue baths of ice-cold bicarbonate buffer solution aerated with a gas composed of 95% O_2_ and 5% CO_2_, with a resulting pH of 7.4 [23].

B. Human samples of intracranial MMA branches were acquired from patients undergoing neurosurgery at the University Hospital of Lund and the Erasmus University Medical Center (Rotterdam, the Netherlands). The vessel segments themselves were from visually healthy tissue.

The artery samples were upon removal immediately placed in cold Dulbecco’s modified Eagle’s medium (DMEM, Gibco, Invitrogen, Carlsbad, CA, USA) in Lund and in cold medium M199 (Gibco, Invitrogen, Carlsbad, CA, USA) in Rotterdam and immediately transported to the laboratory where they were prepared in an identical way to the rat MMA.

### Myography

Each segment of MMA was mounted on a pair of thin metal wires (human: 40 μm, rat: 20/25 μm) in an arterial myograph. One wire was connected to a micrometer screw, allowing for fine adjustment of the vascular tone by varying the distance between the wires. The other wire was connected to a force displacement transducer, paired with an analogue-digital converter (ADInstruments, Oxford, UK). Data was recorded on a computer using a PowerLab unit (ADInstruments).

The aerated bicarbonate buffer was heated to +37°C and used to submerge the wires with the MMA-segment during the experiment. The segment was normalized, attaining 90% of the internal circumference that a fully relaxed vessel would have under a transmural pressure of 100 mmHg or 50 mmHg (see below). A reference value for the contractile capacity of the segment was determined by temporarily replacing part of the NaCl in the buffer solution with 60 mM K^+^.

The effects on the segment of the different vasoactive substances was tested by first recording the spontaneous resting diameter of the artery segment and define it as the maximum dilatation possible. Next, the segment was pre-contracted with 10^-6^ M prostaglandin F_2α_ (PGF_2α_) to achieve a stable tension for the duration of the experiment. Finally, concentrations of the tested substances in the range of 10^-10^-10^-5^ M (the exact range varied between substances) were applied cumulatively and the vessel response was recorded. Because of the unstable response to PGF_2α_ in the first rat experiments, we changed the protocol and used 30 mM K^+^ as a more stable contraction. In addition, the transmural pressure was lowered to 90% of 50 mmHg in these experiments.

Artery segments were omitted from the study if they failed to fulfill the inclusion criteria. Segments included in the final calculations were required to respond to initial testing with K^+^ and to the pre-contraction with a maximum contractile capacity of at least 1 mN for human arteries and 0.1 mN for rat arteries.

### Studies with clinically tested anti migraine compounds

The vessel segments were precontracted with 30 mM K^+^. After precontraction the vessel segments were dilated with 4 nM CGRP. When a stable dilatation was achieved, sumatriptan or telcagepant was added to the arteries in concentrations corresponding to the C_max_ (160 nM and 5 μM, for the 100 mg and 300 mg oral doses, respectively) [24-26], as well as in concentrations corrected for plasma protein binding. The C_max_ for sumatriptan was corrected for 17.5% plasma protein binding [27], while the C_max_ for telcagepant was calculated using a plasma protein binding of 96.4% [28].

### Calculations

The maximum dilatory response (E_max_(%)) for each tested substance was obtained for the vessel segments as the percentage tonus relaxed by the maximum concentration of substance from the PGF_2α_- or K^+^-induced pre-contraction towards the resting tonus sampled before the lowest concentration of substance was added. In the case of significant relaxation the negative logarithm of the concentration of substance required for dilatation of the vessel segments to half of their E_max_(%). The pEC_50_ was obtained for the purpose of comparing the potency of the different substances, calculated using GraphPad Prism software (GraphPad Software, San Diego, California, USA).

If a test subject contributed with more than one vessel segment to the testing of a substance, all results obtained from that individual regarding that substance were pooled together into an average before further processing of data. The hypothesis was that each substance would induce vasodilatation and to determine the significance of the dilatation an unpaired t test was performed using the GraphPad Prism software (GraphPad Software, San Diego, California, USA.) comparing the average E_max_(%) of the highest concentration of each substance to the average E_max_(%) of NaCl buffer at the end of the session.

**Figure 1:**
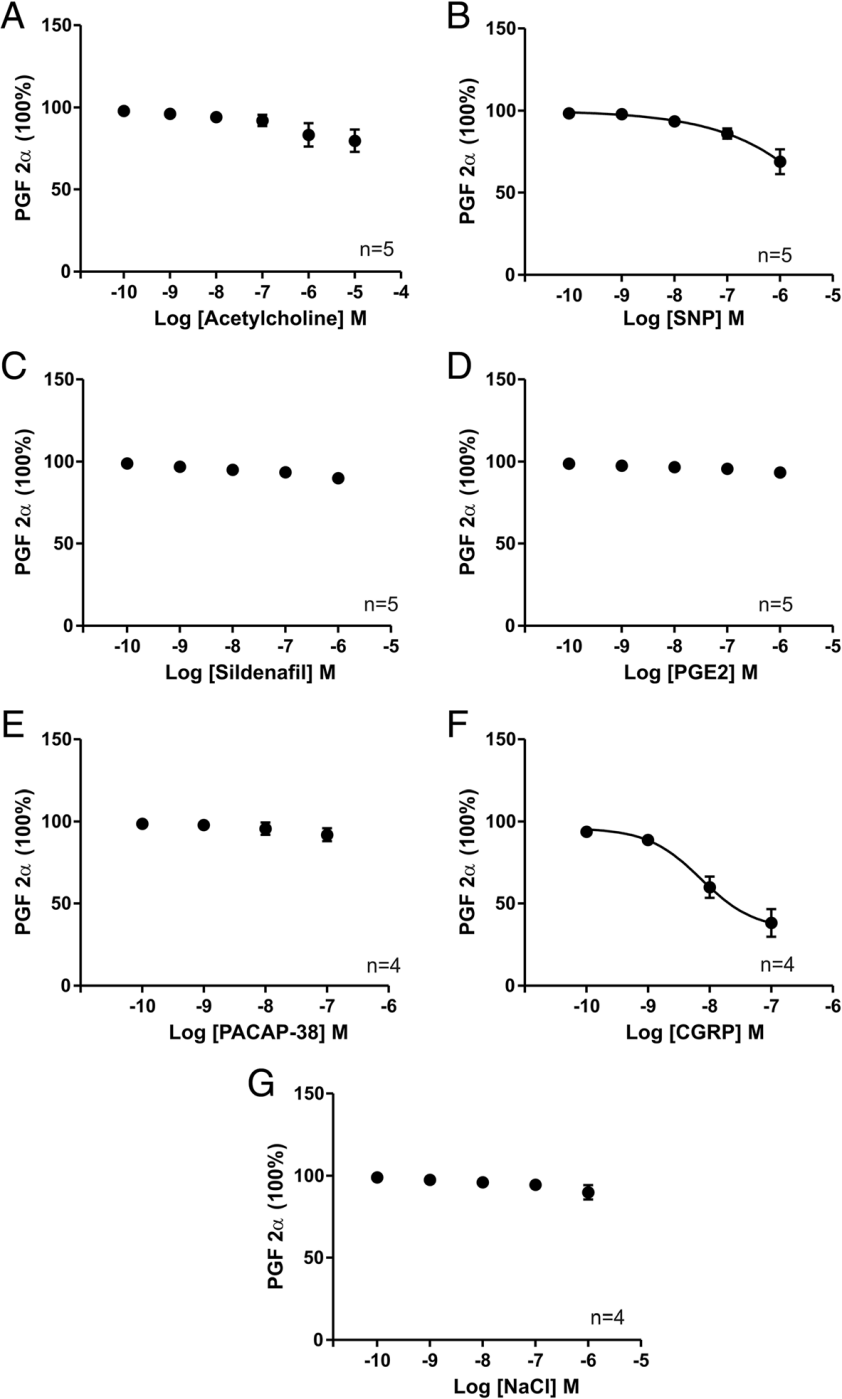
**The effect of substances on human middle meningeal arteries precontracted with PGF**_**2α**_**.** The relaxation relative to PGF_2α_ induced tone in human meningeal arteries by increasing concentrations of each of the tested substances **(A-F)** and NaCl buffer **(G)**. Values are given as mean ± SEM (n = 4 – 6), where PGF_2α_ precontraction is set to be 100%.

**Figure 2:**
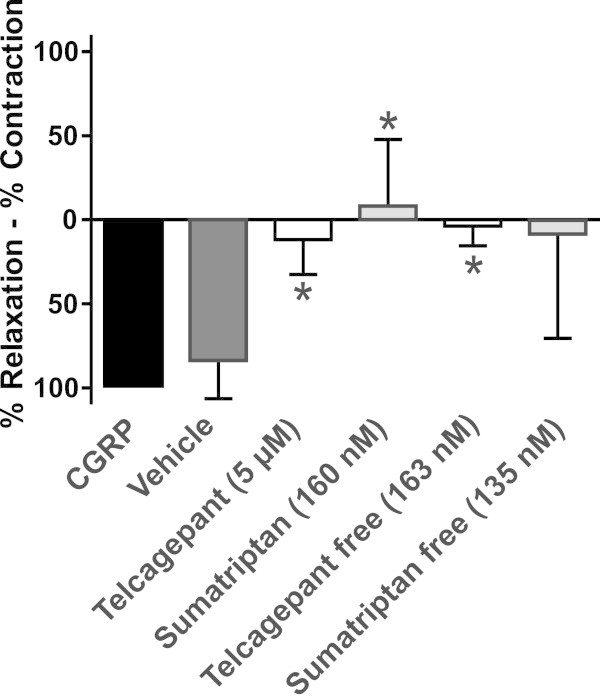
**Effect of telcagepant and sumatriptan on CGRP induced relaxation of the middle meningeal artery.** Arteries were precontracted with 30 mM K^+^, dilated with 4 nM CGRP and sumatriptan or telcagepant was subsequently added (* = p < 0.05). Values are given as % of the CGRP relaxation (mean ± SEM, n = 5).

**Figure 3:**
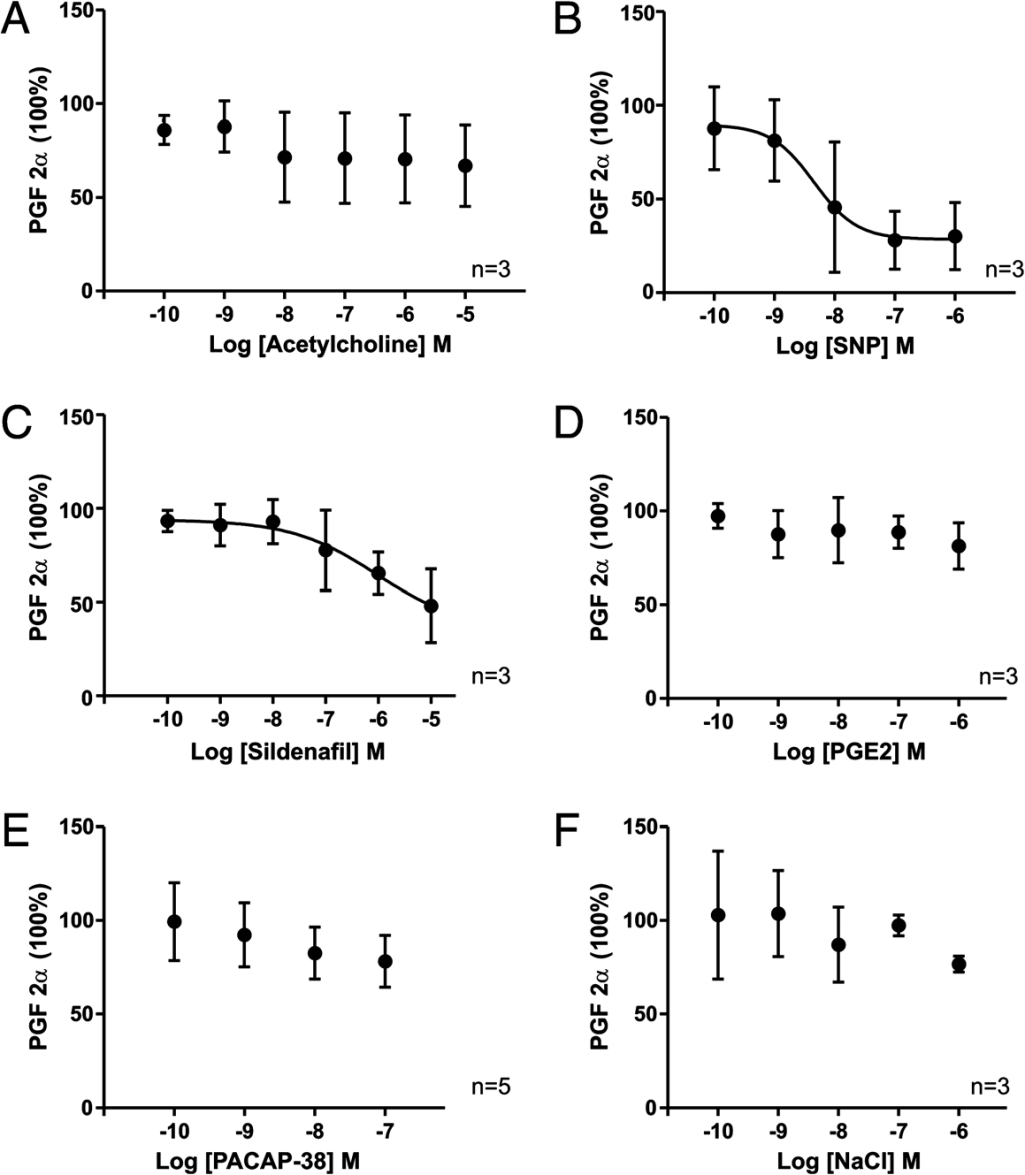
**The effect of substances on rat middle meningeal arteries precontracted with PGF**_**2α**_**.** The relaxation induced in rat meningeal arteries pre-contracted with PGF_2α_ at increasing concentrations of each of the tested substances **(A-E)** and NaCl buffer **(F)**. Values are given as mean ± SEM (n = 3 – 5), where PGF_2α_ precontraction is set to be 100%.

**Figure 4:**
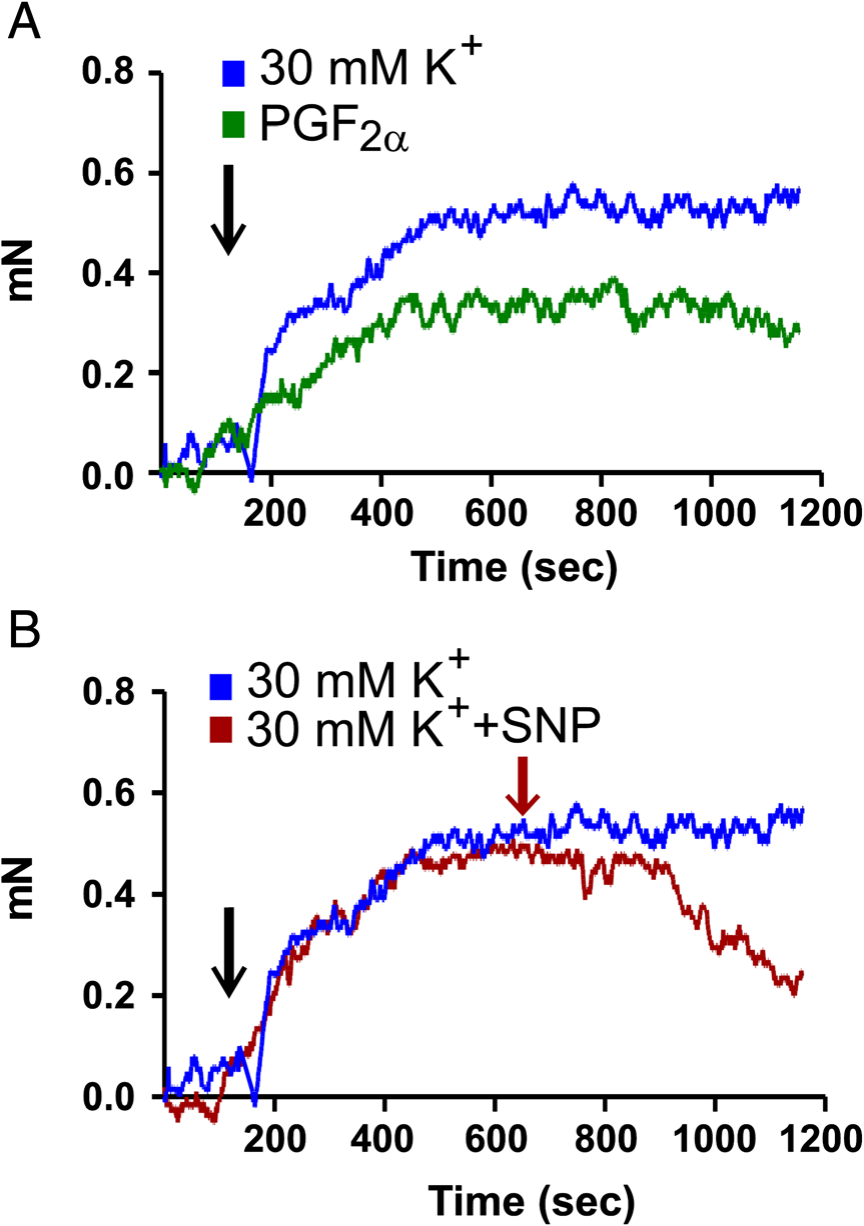
**Comparison of 30 mM K**^**+ **^**and PGF**_**2α **_**on rat middle meningeal arteries, and sample relaxation. A)** Sample contraction induced by of 30 mM K^+^ or PGF_2α_ in rat meningeal artery. One can observe the slight positive drift for 30 mM K^+^ and slight the negative drift for PGF_2α_. **B)** Sample relaxation in an artery precontracted with 30 mM K^+^ and the cumulative relaxation of added sodium nitroprusside (SNP). Small plateaus were allowed before next concentration was added.

**Figure 5:**
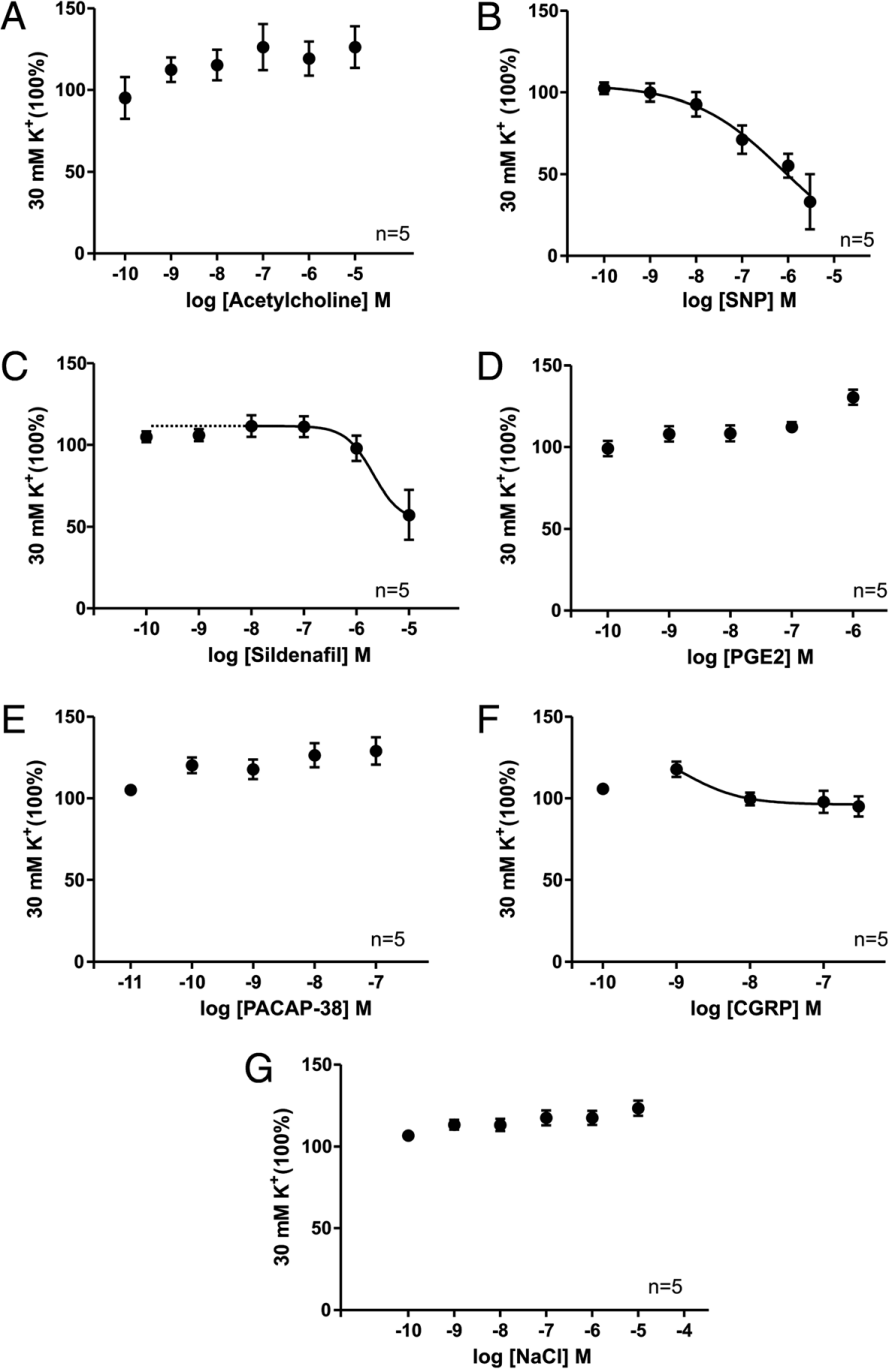
**The effect of substances on rat middle meningeal arteries precontracted with K**^**+**^**.** The relaxation induced in rat meningeal arteries pre-contracted with K^+^ at increasing concentrations of each of the tested substances **(A-F)** and NaCl buffer **(G)**. Values are given as mean ± SEM (n = 5), where 30 mM K^+^ precontraction is set to be 100%).

Figures were compiled where it is shown how the PGF_2α_/K^+^ precontraction (100%) during a session is restored towards the original resting baseline at different concentrations of each of the substances tested and NaCl buffer (Figures 1, 2, 3, 4, and 5). The contractile responses to telcagepant and sumatriptan were expressed as percentage of the previous relaxant response to 4 nM CGRP in the same segment (Figure 2). All data are presented as mean ± S.E.M. Statistical analysis and pEC_50_ calculations were performed using Graphpad Prism 5 software. Statistical significance was accepted at P < 0.05.

### Chemicals

The vasoactive substances used in the present study were: acetylcholine chloride (ACh), sodium nitroprusside (SNP), sildenafil, PACAP-38 (all purchased from Sigma Aldrich), PGE_2_ (Larodan fine Chemicals AB). The inhibitors used were the following: Telcagepant (MK-0974) (dissolved in DMSO and further diluted in H_2_O) (MSD, Whitehouse Station, NJ, USA), sumatriptan (dissolved in H_2_O) (Sigma Aldrich).

## Results

### Functional results from the MMA

Intracranial MMA segments from 6 humans were used to test the vasoactive properties of six different compounds including NaCl buffer but excluding CGRP. The vessels were divided into several segments of 1–2 mm in length and studied in parallel tissue baths. Not every segment was exposed to all compounds. Vessels from 4 additional human patients were used for the testing of CGRP.

### Human MMA (100 mmHg, pre-contracted with PGF_2α_)

Of the compounds tested, SNP and CGRP on human artery showed significant dilatation compared to NaCl, with CGRP being the strongest vasodilator (Figure 1, Table 1). ACh showed a non-significant tendency of relaxation. PGE_2_, PACAP-38 and sildenafil showed no vasomotor activity distinct from the gradual loss of pre-contraction seen when using only NaCl buffer (Figure 1, Table 1). The very high concentration of sildenafil (10 μM) was not tested on human vessels.

**Table 1:** **Human MMA, 100 mmHg, pre-contracted with PGF**_**2α**_

**Compound**	**E**_**max**_**(%)**	**pEC**_**50 **_**(M)**	**p - value**
ACh (n = 6)	17 ± 6	N.A.	0.44
SNP (n = 6)	32 ± 6	~3.5	0.034
Sildenafil (n = 6)	11 ± 2	N.A.	0.91
PGE_2_ (n = 6)	8 ± 1	N.A.	0.62
PACAP-38 (n = 5)	9 ± 3	N.A.	0.89
CGRP (n = 4)	62 ± 8	8.1 ± 0.19	0.0015
NaCl (n = 4)	10 ± 4	N.A.	

p-value for comparing agonist versus the NaCl effect. N.A. = Not Applicable. Values are ± SEM, n given in parenthesis.

### Human MMA and CGRP (100 mmHg, pre-contracted with 30 mM K^+^)

For human samples the clinically tested anti migraine compounds were applied to the isolated arteries that that had been precontracted with 30 mM K^+^. CGRP induced a significant vasodilatation of meningeal arteries (84 ± 10% of precontraction with 30 mM K^+^). The concentrations corresponding to the C_max_ obtained after oral administration of 100 mg sumatriptan (160 nM) or 300 mg telcagepant (5 μM) both lead to a significant decrease of the effect of the exogenous administrated CGRP (-8 ± 18% and 8 ± 8%, respectively) (Figure 2). Also the concentrations of both sumatriptan and telcagepant corrected for plasma protein binding abolished the effect of exogenous CGRP (8 ± 36% for sumatriptan and 4 ± 7% for telcagepant, respectively) (Figure 2).

### Rat MMA, (100 mmHg, pre-contracted with PGF_2α_)

SNP and sildenafil showed a tendency to relax the arteries but the observed dilatation was not statistically significant (Figure 3, Table 2). ACh, PGE_2_ and PACAP-38 did not display any discernible vasodilatation compared to NaCl (Figure 3, Table 2). The results from these rat experiments showed a larger spread compared to the experiments using human artery segments or a different protocol, and thus had a higher ratio of not passing the inclusion criteria.

**Table 2:** **Rat MMA, 100 mmHg, pre-contracted with PGF**_**2α**_

**Compound**	**E**_**max**_**(%)**	**pEC**_**50 **_**(M)**	**p - value**
ACh (n = 3)	33 ± 22	N.A.	0.68
SNP (n = 3)	70 ± 18	8.3 ± 0.7	0.066
Sildenafil (n = 3)	52 ± 20	~7	0.23
PGE_2_ (n = 3)	23 ± 12	N.A.	0.82
PACAP-38 (n = 4)	22 ± 14	N.A.	0.93
NaCl (n = 3)	23 ± 4	N.A.	

p-value for comparing agonist versus the NaCl effect. N.A. = Not Applicable. Values are ± SEM, n given in parenthesis.

For the first experiments above, the same protocol as for the human vessels (100 mmHg and pre-contracted with PGF_2α_) was used to test all substances including NaCl on MMA segments from 5 rats. These vessels showed a large variation and had a high ratio of not passing the inclusion criteria of 0.1 mN. Therefore, the protocol was changed by lowering the transmural pressure to 50 mmHg and the experiments were repeated using an additional 5 rats. This markedly improved the success rate. In addition, the MMAs pre-contracted with 30 mM K^+^, did not have a negative drift over time (Figure 4A). A sample relaxation to a cumulative dose of SNP is also shown (Figure 4B).

### Rat MMA, (50 mmHg, pre-contracted with 30 mM K^+^)

The results showed significant relaxation upon exposure to SNP, CGRP and sildenafil compared to the NaCl control group, SNP being the strongest vasodilatator (Figure 5, Table 3). PGE_2_, ACh and PACAP-38 showed no significant response compared to the control group (Figure 5, Table 3). These results correlate with the tendency seen for the above MMA.

**Table 3:** **Rat MMA, 50 mmHg, pre-contracted with 30 mM K**^**+**^

**Compound**	**E**_**max**_**(%)**	**pEC**_**50 **_**(M)**	**p - value**
ACh (n = 5)	-26 ± 13	N.A.	0.85
SNP (n = 5)	67 ± 22;	6.1 ± 1.3	0.015
Sildenafil (n = 5)	40 ± 17	5.7 ± 3.8	0.0069
PGE_2_ (n = 5)	-31 ± 5	N.A.	0.311
PACAP-38 (n = 5)	27 ± 8	N.A.	0.94
CGRP (n = 5)	-1 ± 6	~ 8	0.0015
NaCl (n = 5)	-24 ± 5	N.A.	

p-value for comparing agonist versus the NaCl effect. N.A. = Not Applicable. Values are ± SEM, n given in parenthesis.

## Discussion

The addition of substances on human vessels was studied at a transmural pressure of 100 mmHg and pre-contracted with PGF_2α_. Two different approaches for the rat MMAs were used, and in this article the results from both methods are presented. Lowering of the transmural pressure from 100 mmHg to 50 mmHg greatly improved the success rate, and the more constant 30 mM K^+^ pre-contraction improved reproducibility. The gradual failing of the PGF_2α_ pre-contraction and increase of the K^+^-induced pre-contraction was mitigated by comparing the vasomotor activity of the substances with the spontaneous activity observed when subjecting the vessel segments to only NaCl buffer. It is important to inform the reader, that the isolation and insertion of wires into the rat MMA, destroys the endothelial cell layer because of the small diameter of the rat MMA (around 100 μm in o.d.). The lack of endothelium can be seen in Figure 4A because ACh induces a contraction, most likely acting on ACh receptors on the smooth muscle cells. The endothelial function in the human vessels is not substantial either, as no significant relaxation to ACh is observed. The lack of full endothelial function and the lack of sympathetic tone in the vessels in this study could alter some of the effects of the substances added. For example, sildenafil has both endothelial-dependent and -independent effects [29], which could be different in human and rat.

The results from both types of experiments on rat tissue were consistent and showed that SNP, CGRP and sildenafil induced vasodilatation, while ACh, PGE_2_ and PACAP-38 had little effect. When used on human vessels, the same myography method yielded significant results for SNP and CGRP, where a strong dilatation was observed. An alternative method that has been used in rodents with some success is the cranial window method where substances can be applied topically through a thinned calvarium [30], but with this method quantification is difficult, as the concentration of active substance that reaches the vessel is difficult to control, hence pEC_50_ and E_max_ cannot reliably be calculated. Further, there are more factors (e.g., blood pressure) that can influence the results than when using isolated vessels *in vitro*.

For the experiments on human vessels, the greatest difference in response between MMA and the previously published data for MCA [20] was seen when subjecting the vessels to sildenafil and PACAP-38, which in both cases constituted a stronger dilatation in the MCA. This was surprising in the case of sildenafil since there are previous reports of rat MMA containing higher levels of phosphodieterase type 5 (PDE5) than reported for rat MCA [31]. If the same conditions were valid for human vessels, the higher concentration of the target enzyme in the MMA means that sildenafil should have had more effect there. However, the effect is also dependent on basal NO and cGMP levels, and we cannot rule out that there could be an influence of the age of the human compared to the rat vessels. In addition, the sympathetic tone *in vivo* could be counteracted by increased NO production, which would not occur in our setup. Therefore, sildenafil could be effective *in vivo*, but not in the myographs. It is hard to draw conclusions about the difference in E_max_(%) of PACAP-38 due to previous conflicting observations of the activity of PACAP-38 in rat. Previously published *in vitro* experiments where human intracranial MMA was subjected to the substance suggests that PACAP-38 could indeed dilate human MMA (E_max_(%) = 34 ± 12; pEC_50_ = 6.9 ± 0.1) [32] and that the absence of vasodilatation observed in the rat experiments in the present study is either due to lack of receptors or technical issues. However, there is no data suggesting an endothelial response to PACAP-38. This suggests that the rat is not a good model for studies of the PACAP-38 mechanisms.

Previous *in vivo* studies exist where magnetic resonance angiography (MRA) was used to measure the diameter of the extracranial part of the MMA of live human test subjects before, during and after spontaneous and artificially induced episodes of migraine. Intravenous infusion of CGRP [33], PACAP-38 [34] and nitroglycerine [35] in healthy non-migraineurs were shown to increase the diameter of the extracranial MMA. These substances are known to induce a headache lacking the migraine-related characteristics in non-migraineurs and this type of pain coincided with the observed vascular dilatation. When CGRP was infused to migraineurs, the results were reproduced and an additional, migraine-like headache with delayed onset occurred [36]. On the other hand, spontaneous migraine attacks reported in patients were associated with a modest increase in diameter (a magnitude of 11.4-13.0%) of the MCA and the intracranial parts (cavernous and cerebral) of the ICA [37]. The intracranial basilar artery (BA) and the extracranial vessels of ECA, superficial temporal artery (STA), the cervical part of the ICA and the extracranial part of the MMA were not significantly dilated.

Migraine-aborting triptan drugs had a constricting effect on arteries outside of the BBB, like the MMA and the extracranial part of the ICA, but not on cerebral arteries including the intracranial part of the ICA. Regardless of whether an episode of migraine was triggered naturally or by an infused substance, the pain was mitigated and the extracranial MMA contracted from the use of triptan drugs. However, it should be noted that these studies did not include the intracranial branches of the MMA, which is the source of the vessel segments used presently in the *in vitro* studies, and it is still unknown how this part of the artery would be affected *in vivo* by a spontaneous episode of migraine or a therapeutic dose of sumatriptan. The question is relevant, because all arteries shown to significantly dilate during an episode of migraine are all intracranial. Intracranial meningeal arteries would be unique in that they are both intracranial and without BBB properties. They are thereby a type of artery that could possibly be both dilated during an episode of migraine and contracted by drugs. In the *in vitro* study presented here, the effect of two well-known clinically tested anti migraine compounds were applied to the isolated human MMAs. Both sumatriptan (5-HT receptor agonist) and telcagepant (a CGRP receptor antagonist) significantly reduced the CGRP induced vasodilation. This shows that the vasoactive clinically tested compounds can exert their effect directly on the MMA.

*In vivo* experiments in rat, using the cranial window method to directly view and apply substances to the intracranial portion of the MMA, have yielded similar results to the *in vivo* observations in human regarding the extracranial part of the vessel. The MMA of rats was significantly dilated by α- and βCGRP [38,39], sildenafil [31], VIP, PACAP-38, PACAP-27 [40], histamine [41] and ACh [42]. VIP is interesting in that it is not a migrainogenic substance [16]. It has not been established what effect VIP could have on human isolated MMA *in vivo*, but since VIP is known to dilate human MMA branches *in vitro*[32] one can assume that the *in vivo* effect would mirror that found in the rat. Vasodilatation without migraine-related pain speaks against the dilation of the MMA as a direct cause of the pain. As with the effect of other substances on meningeal arteries, there are conflicting reports regarding VIP. No significant dilatation was observed during *in vitro* experiments using the myography method on the MMA [43]. Using the pressurized arteriography method cerebral and MMA were found to relax to the addition of VIP and PACAP [44,45]. PACAP was more potent than VIP [44] while the reverse was seen in the cerebral artery [45].

When the vasomotor responses from our experiments are compared with the migrainogenic properties of the tested substances, there seems to be little correlation between the ability of a substance to induce migraine-like attacks and their vasoactive properties in human meningeal arteries. Sildenafil, PGE_2_ and PACAP-38 showed no tendency of dilatation of the arteries in the current study, despite having known migrainogenic properties. One may wonder why and how the migrainogenic substances induce headache if vasodilatation of meningeal or cerebral arteries is not the mechanism. Endothelial nitric oxide (NO) is not the key molecule since the intracellular pathways activated by the migrainogenic substances CGRP and PACAP-38 are not dependent of NO in the cranial vessels [20]. The remaining factors are the perivascular and dural nerve fibers. It has been shown before that the dura mater contains a rich supply of sensory substance P and CGRP fibers, and of sympathetic noradrenaline and neuropeptide Y, but only a minor amount of parasympathetic VIP and PACAP containing fibers [46]. It is tentative to suggest that local release or systemic administration of the various substances may modify the activity of the dural nerve fibers via specific receptors or mechanisms. This clearly deserves future attention.

## Conclusion

The lack of correlation between the vasoactive and migrainogenic properties of the tested substances leads us to the conclusion that direct vasodilatation of intracranial meningeal arteries is most likely not the sole trigger of the artificial migraine-like pain experienced by migraine sufferers upon infusion of these substances into the blood stream. This opinion is based on the fact that several migrainogenic substances induced no significant vasodilatation when applied *in vitro* to human meningeal arteries. We can however not rule out that the cranial pain and migrainogenic properties associated with the infusion of these substances could have a relation to cranial vasodilation *in vivo.* The *in vivo* vasodilatory responses to the substances could be affected by the vascular tone, endothelial interplay and the activation of perivascular nerve fibers around the dural arteries or in the trigeminal ganglion.

## Competing interests

The authors declare that they have no competing interests.

## Authors’ contributions

Conceived and designed the experiments: GG, SL, KAH, AMvdB, LE. Performed the experiments: GG, SL, KAH. Analyzed the data: GG, SL, KAH, AMvdB. Contributed reagents/materials/analysis tools: AMvdB, LE. Wrote the paper: GG, SL, KAH, AMvdB, LE. All authors read and approved the final manuscript.
